# Characterizing Whole Brain Temporal Variation of Functional Connectivity via Zero and First Order Derivatives of Sliding Window Correlations

**DOI:** 10.3389/fnins.2019.00634

**Published:** 2019-06-27

**Authors:** Flor A. Espinoza, Victor M. Vergara, Eswar Damaraju, Kyle G. Henke, Ashkan Faghiri, Jessica A. Turner, Aysenil A. Belger, Judith M. Ford, Sarah C. McEwen, Daniel H. Mathalon, Bryon A. Mueller, Steven G. Potkin, Adrian Preda, Jatin G. Vaidya, Theo G. M. van Erp, Vince D. Calhoun

**Affiliations:** ^1^Mind Research Network, Albuquerque, NM, United States; ^2^Tri-Institutional Center for Translational Research in Neuroimaging and Data Science (TReNDS), Georgia State University, Georgia Institute of Technology, Emory University, Atlanta, GA, United States; ^3^Department of Mathematics and Statistics, The University of New Mexico, Albuquerque, NM, United States; ^4^Department of Electrical and Computer Engineering, The University of New Mexico, Albuquerque, NM, United States; ^5^Department of Psychology and Neuroscience, Georgia State University, Atlanta, GA, United States; ^6^Department of Psychiatry, University of North Carolina at Chapel Hill, Chapel Hill, NC, United States; ^7^Department of Psychiatry, University of California, San Francisco, San Francisco, CA, United States; ^8^San Francisco VA Medical Center, San Francisco, CA, United States; ^9^Pacific Neuroscience Institute, Santa Monica, CA, United States; ^10^John Wayne Cancer Institute, Department of Translational Neurosciences and Neurotherapeutics, Santa Monica, CA, United States; ^11^Department of Psychiatry, University of Minnesota, Minneapolis, MN, United States; ^12^Department of Psychiatry and Human Behavior, University of California, Irvine, Irvine, CA, United States; ^13^Department of Psychiatry, The University of Iowa, Iowa City, IA, United States; ^14^Clinical Translational Neuroscience Laboratory, Department of Psychiatry and Human Behavior, University of California, Irvine, Irvine, CA, United States; ^15^Center for the Neurobiology of Learning and Memory, University of California, Irvine, Irvine, CA, United States

**Keywords:** functional network connectivity, group independent component analysis, windowed correlation, derivatives, resting state fMRI

## Abstract

Brain functional connectivity has been shown to change over time during resting state fMRI experiments. Close examination of temporal changes have revealed a small set of whole-brain connectivity patterns called dynamic states. Dynamic functional network connectivity (dFNC) studies have demonstrated that it is possible to replicate the dynamic states across several resting state experiments. However, estimation of states and their temporal dynamicity still suffers from noisy and imperfect estimations. In regular dFNC implementations, states are estimated by comparing connectivity patterns through the data without considering time, in other words only zero order changes are examined. In this work we propose a method that includes first order variations of dFNC in the searching scheme of dynamic connectivity patterns. Our approach, referred to as temporal variation of functional network connectivity (tvFNC), estimates the derivative of dFNC, and then searches for reoccurring patterns of concurrent dFNC states and their derivatives. The tvFNC method is first validated using a simulated dataset and then applied to a resting-state fMRI sample including healthy controls (HC) and schizophrenia (SZ) patients and compared to the standard dFNC approach. Our dynamic approach reveals extra patterns in the connectivity derivatives complementing the already reported state patterns. State derivatives consist of additional information about increment and decrement of connectivity among brain networks not observed by the original dFNC method. The tvFNC shows more sensitivity than regular dFNC by uncovering additional FNC differences between the HC and SZ groups in each state. In summary, the tvFNC method provides a new and enhanced approach to examine time-varying functional connectivity.

## Introduction

Connectivity studies have uncovered a complex functional organization of brain connectome thanks to the use of functional magnetic resonance imaging (fMRI) (Fox et al., 2005; Power et al., 2011). The existence of disease-related abnormalities in the human connectome brings progress toward the use of fMRI acquisition in the clinical setting (Fox et al., 2010). As with any biological system, the brain connectome does not function in a static manner. Researchers have recognized the importance of developing techniques to characterize dynamic features embedded in the connectome dynamics (Hutchison et al., 2013; Saggar et al., 2018). Although one of the most basic measures of dynamicity is the derivative, this feature is underexplored in the context of functional connectivity. This limitation is related to the fact that functional connectivity is linked to the phase between neuronal activations (Yaesoubi et al., 2015). Study of the phase dynamics is more difficult to characterize than the dynamics of the actual activations. This work fills the gap by focusing on the derivative as a measure of the instantaneous variation of brain connectivity.

Functional connectivity measures the level of co-activation of fMRI time-series from anatomically separated brain regions (Friston et al., 1993). Previous connectivity studies considered functional connectivity to remain constant during the scan duration (Allen et al., 2011; Espinoza et al., 2018). Recent studies applying the dynamic FNC method (dFNC) have demonstrated that temporal functional network connectivity (FNC) analysis (i.e., co-activation between covarying networks estimated via independent component analysis) can uncover reoccurring connectivity patterns at resting state or during task performances. Their results also indicate that brain connectivity patterns iterate through time and show smooth variations of connectivity (Allen et al., 2014; Calhoun et al., 2014; Damaraju et al., 2014; Rashid et al., 2014; Espinoza et al., 2019). The dFNC method provides a way to explore temporally transient changes in the functional connectivity among brain networks using sliding windows to compute FNC across time (Sakoğlu et al., 2010; Allen et al., 2014). Among the limitations of the dFNC method, is the lack of justification for the reporting of very similar connectivity states identified by k-means clustering algorithm. At glance, the similar states can be combined into one state without taking into account the states temporal behavior. In this work, we aim to improve the ability of the dFNC method to characterize connectivity dynamics by including derivatives of windowed FNC in the identification of reoccurring states of connectivity.

Our approach referred to as temporal variation of functional network connectivity (tvFNC) is validated with a simulated data sample, and then applied to a resting-state fMRI sample formed by healthy controls (HC) and schizophrenia (SZ) patients that was previously analyzed with the original dFNC method (Damaraju et al., 2014). Among our goals were: to extend time-varying FNC states characterization by including the first derivatives of the windowed FNC; to provide complement states differentiation by including their derivatives information; to expose group differences not captured by the current dFNC method.

## Methods

### Static Functional Network Connectivity

**FIGURE 1:**
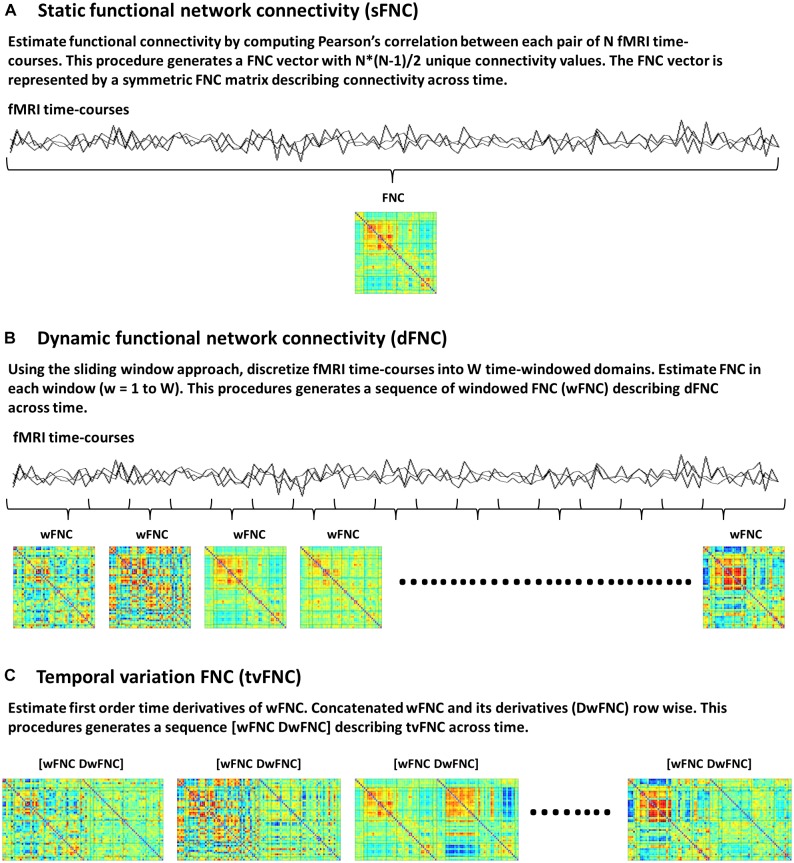
Functional network connectivity (FNC) subject’s data, **(A)** Static FNC, **(B)** Dynamic FNC, and **(C)** Temporal variation of FNC.

Static FNC (sFNC) analysis is based on the assumption that functional connectivity, defined as statistical dependence among N number of fMRI time-courses does not change during scanning time. Currently available connectivity measures include correlations (Rodgers and Nicewander, 1988), coherence (Chang and Glover, 2010; Yaesoubi et al., 2015) and mutual information (Gomez-Verdejo et al., 2012; Wang et al., 2015) among others. In this study, functional connectivity is measured via the Pearson’s pairwise correlation, which is the most widely used approach to date (Allen et al., 2011; Espinoza et al., 2018). Correlations between each pair of time-courses generates a FNC vector with N^∗^(N-1)/2 unique FNC values. The FNC vector is then represented by an NxN symmetric FNC matrix (Figure 1A).

### Dynamic Functional Network Connectivity

The dFNC analysis is an extension of sFNC, developed to capture time-varying FNC. In this method each time-course is discretized into a set of time domains using the sliding windowed approach (Sakoğlu et al., 2010; Allen et al., 2014). Then, in each time-windowed domain a FNC vector is calculated. This procedure generates a discrete sequence of windowed FNC (wFNC) vectors that are then represented by wFNC matrices (Figure 1B) describing connectivity behavior across time (Sakoğlu et al., 2010; Allen et al., 2014; Damaraju et al., 2014; Rashid et al., 2014; Espinoza et al., 2019). Subjects’ dFNC data is formed by all wFNC vectors, and is referred to as the zero order derivatives of the sliding window correlations. In summary, the dFNC method pipeline is as follows, for all subjects compute sliding windowed correlations (wFNC); form dFNC data by stacking time-wise all subjects’ dFNC data; run clustering analysis on dFNC data to identify reoccurring connectivity states.

### Temporal Variation of Functional Network Connectivity

The tvFNC analysis is an extension of dFNC, aiming to improve state classification by including wFNC derivatives in the clustering step. First order time derivatives of wFNC vectors are computed using finite difference approximations. For each subject, the discrete derivative of the first wFNC was estimated using the forward difference formula, Dw_1_FNC = w_2_FNC – w_1_FNC. The discrete derivatives of the interior wFNC were estimated using central difference formula, Dw_i_FNC = (w_*i*+1_FNC – w_*i*−1_FNC)/2, for *i* = 2 to W−1, where W is the number of windows. Lastly, the discrete derivative of the last wFNC was estimated using the backward difference formula, Dw_W_FNC = w_W_FNC – w_W−1_FNC. Subjects’ DdFNC data is formed by all wFNC derivatives, and is referred to as the first order derivatives of the sliding window correlations.

The tvFNC method pipeline is as follows, for all subjects (1) compute dFNC data (sliding windowed correlations wFNC); (2) estimate DdFNC data (derivatives of sliding windowed correlations DwFNC); (3) concatenate row wise zero and first order windowed correlations [wFNC and DwFNC] divided by their corresponding standard deviations (Figure 1C). The tvFNC data is formed by stacking time-wise all subjects [dFNC and DdFNC] data, and is referred to as the zero and first order derivatives of the sliding window correlations; (4) run clustering analysis on all subjects’ tvFNC data to identify reoccurring connectivity states and their derivatives patterns.

### Clustering Analysis

In both methods dFNC and tvFNC, time-varying connectivity is captured by performing k-means clustering analysis, assigning all subjects’ temporal FNC data into a selected number of clusters representing distinct functional connectivity states. The clustering algorithm selection is based on previous connectivity studies that successfully applied k-means algorithm to identify reoccurring patterns of connectivity within and between subjects across time (Allen et al., 2014; Calhoun et al., 2014; Damaraju et al., 2014; Rashid et al., 2014; Faghiri et al., 2018; Vergara et al., 2018; Espinoza et al., 2019). We refer to Allen et al. (2014), Damaraju et al. (2014), and Abrol et al. (2017) for details on k-means clustering validation. The k-means clustering algorithm is applied to the temporal FNC data with the number of clusters ranging from 1 to K along with the Elbow criterion to identify the optimal number of clusters referred to as states. The optimal number of clusters is selected from the Elbow criterion cluster index results. The cluster index is defined as the ratio between the sums of the within-cluster sums of point-to-centroid distances to the sums of all the distances from each point to every centroid (Allen et al., 2014).

### Simulated Data

**FIGURE 2:**
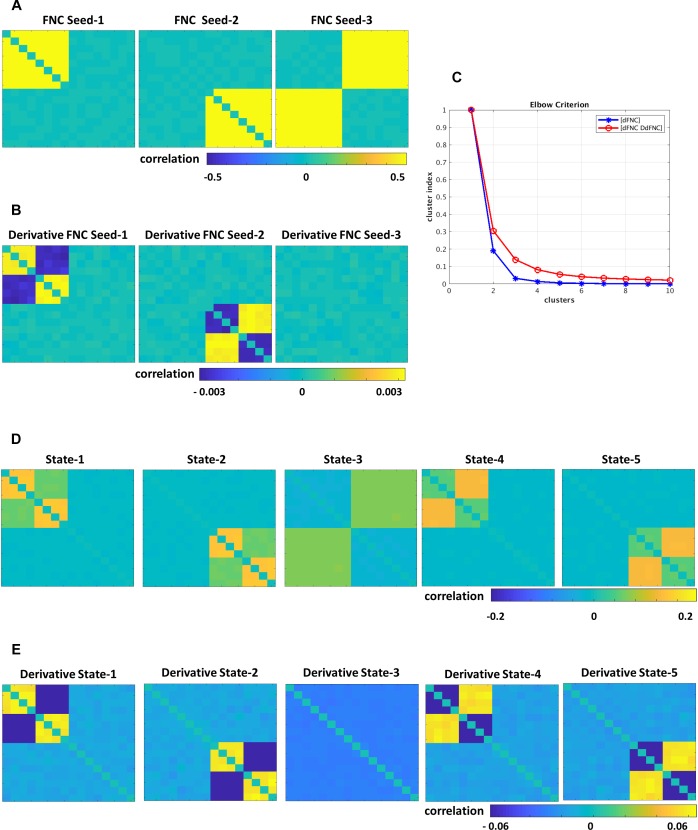
Simulated data, **(A)** FNC seeds, **(B)** derivatives of FNC seeds, **(C)** Elbow criterion results for dFNC and tvFNC methods, panels **(D,E)** show FNC states and their derivatives choosing optimal number of clusters = 5.

The simulated data was designed to show the tvFNC method for a given a number of subjects S, and their corresponding N number of fMRI time-courses. For simplicity, we considered *N* = 12 and generated tvFNC data for *S* = 300 subjects. For simulation purposes, the subjects were divided into five groups with the same number of individuals in each group. For each subject, a time-varying sequence of 136 wFNC vectors describing subjects’ dFNC behavior during scan duration was created. The number of windows, *W* = 136 was chosen to match the one obtained in the dFNC analysis of the selected fMRI data sample. Each simulated FNC and wFNC vectors contains 66 [=N^∗^(N−1)/2)] unique FNC pairs. Subjects’ dFNC data sets were created using three distinct connectivity patterns referred to as FNC seeds plus perturbation seeds created using random noise and white Gaussian noise (σ = 0.003). The first FNC seed shows a pattern with positive connectivity in the upper left block. The second FNC seed shows a pattern with positive connectivity in the lower right block. The third FNC seed shows a pattern with positive connectivity in the upper right and lower left blocks. FNC seed patterns are shown in Figure 2A. The perturbation seeds were chosen to be the first derivatives of the FNC seeds. These derivatives were tailored to have unique patterns simulating subject specific differences existing in real data. FNC seed derivatives patterns are shown in Figure 2B.

The simulation is initialized by setting the first window to the seed pattern plus noise: w_1_FNC = FNC seed. Windowed FNC vectors are then simulated by using the recursive equation: w_*i*+1_FNC = w_i_FNC ± DFNC seed + N(0,σ), *i* = 1 to W−1. The symbol ± indicates that DFNC seed was added in some subjects, but subtracted in others. The recurrent equation was applied only to generate dFNC data from seeds 1 and 2. Dynamic FNC data generated from the third FNC seed did not include the derivative term DFNC seed. This way we covered the cases where states can have different derivative patterns across time (derivatives could go in opposite directions) and where there are no significant derivative changes. Next, first order time DdFNC of simulated data were computed, and tvFNC data was formed as previously explained. Then, the k-means clustering algorithm was applied to each simulated temporal FNC set ([dFNC] and [dFNC DdFNC]) with the number of clusters ranging from 1 to 10 along with the Elbow criterion to identify the optimal number of clusters. The simulations were repeated one hundred times and mean values of cluster index were computed.

### Resting State fMRI Data

#### Data Sample

The resting state functional magnetic resonance imaging (fMRI) data used in this study was taken from the Functional Imaging Biomedical Informatics Research Network (FBIRN) Phase III study. Participants (healthy controls and patients) were recruited in seven sites across the United States. Participants’ information and scan collection was approved by all seven sites’ institutional review boards (IRB). The sample is formed by a total of 314 participants. The cohort includes 163 healthy controls (117 males, 46 females; average age 36.9 years) and 151 age- and gender matched patients with SZ (114 males, 37 females; average age 37.8 years).

#### Data Acquisition

All participants provided written informed consent before scanning. Resting-state fMRI scans were collected at seven sites using a 3T Siemens Tim Trio System scanner in six locations and a 3T General Electric Discovery MR750 scanner in one location. Participants were asked to lay still, stay awake and keep their eyes closed during the whole scan duration. In all sites, T2^∗^-weighted gradient-echo echo-planar images (EPIs) were acquired with the following parameters: voxel size = 3.4375 × 3.4375 × 4.0 mm^3^, repetition time (TR) = 2 s, eco time (TE) = 30 ms, flip angle (FA) = 77 degrees, field of view (FOV) = 220 × 220 mm (64 × 64 matrix), slice thickness = 4 mm, gap = 1 mm, number of slices = 32 sequential ascending slices. Scans lasted 5:4 min, a total of 162 volumes of echo planar imaging BOLD fMRI were collected.

#### Data Pre-processing, Group Independent Component Analysis, and Post-processing

Detail information of selected rsfMRI scans quality control, pre-processing, group independent component analysis (GICA), and post-processing can be found in Damaraju et al. (2014). In summary, functional images were preprocessed using custom written Matlab scripts along with three available toolboxes, Analysis of Functional NeuroImages (AFNI)^1^, Spatial Parametric Mapping (SPM)^2^, and Group ICA/IVA of fMRI Toolbox (GIFT)^3^. Rigid body motion correction was performed using INRIalign (Freire and Mangin, 2001). Resting-state fMRI scans were spatially normalized to the Montreal Neurological Institute (MNI) space (Friston, 1995), resliced to 3 mm x 3 mm x 3 mm voxels, and smoothed using a Gaussian kernel with a full-width at half maximum (FWHM) of 6 mm. Lastly, each voxel time-course was variance normalized completing the data preprocess step. Participants (HC and SZ) whole brain functional parcellation was obtained by applying the spatial GICA algorithm implemented in the GIFT toolbox (Calhoun et al., 2001; Correa et al., 2005) to the preprocessed fMRI data. Spatial GICA is an extension of spatial ICA, which decomposes all subjects’ fMRI data into linear mixtures of maximally spatially independent components and provides their unique time-course profiles. One hundred independent components (ICs) representing whole brain functional parcellation were obtained using principal component analysis (Rachakonda et al., 2016) and the infomax algorithm (Bell and Sejnowski, 1995). Subjects’ ICs anatomical brain regions referred as spatial maps and their corresponding time-courses were obtained using the spatiotemporal regression back reconstruction approach (Calhoun et al., 2001; Erhardt et al., 2011). Out of the 100 ICs that were estimated, *N* = 47 ICs were identified as meaningful resting state networks (RSNs) by evaluating the ratio of high to low frequency power in the spectra of components, as well as whether peak activations took place in gray matter (Meda et al., 2008; Robinson et al., 2009; Allen et al., 2011). Post-processing of the selected 47 RSNs time-courses included: detrending and despiking using 3DDespike, filtering using a fifth-order Butterworth low-pass filter with a high frequency cutoff of 0.15 Hz, and variance normalization.

### Estimation of dFNC and tvFNC Data

Whole brain dFNC is computed by obtaining a sequence of time domains for each of the selected 47 RSNs time-courses using the tapered sliding window approach (Allen et al., 2014). A total of 136 widows (*W* = 136) were obtained using a rectangular window width of 22 TRs (=44 s, TR = 2 s) convolved with a Gaussian of sigma 3 TRs, and sliding in one TR step until covering the whole time domain. Then, for each subject’s windows 1 to 136, FNC among the RSN windowed time-courses was calculated generating a discrete sequence of wFNC vectors containing 1081 [=N^∗^(N−1)/2] unique FNC pairs. Each wFNC vector is then represented by a full covariance matrix named wFNC and/or dFNC matrix. Since time-courses of short length may have insufficient information to characterize full covariance matrices, the graphical LASSO algorithm (Friedman et al., 2008) was used to overcome this limitation. Covariance matrices were estimated from regularized inverse covariance matrices (Smith et al., 2011). A penalty on the L1 norm of the precision matrix was applied to enforce sparsity. The cross-validation scheme for estimating covariance with graphical lasso framework is as follows: For each subject, a random windowed data is chosen and rest of the subject’s windowed data is considered as unseen data. The regularization parameter defined as the optimum hyperparameter lambda (among a set of lambda values selected *a priori*) that maximizes the log likelihood of the unseen data is chosen. This process is repeated for few randomly chosen windows of the subject and the mean lambda across the repetitions is then used for estimating covariance for all of the windows of that subject. Overall 42,704 (=314 participants times 136 wFNC) dFNC matrices were obtained representing subjects’ FNC as a function of time. To account for nuisance effects, subjects’ dFNC data (zero order sliding windowed correlations) were Fisher z transformed, and residualized with respect to age, gender and multi-site (Damaraju et al., 2014). Next, time derivatives of the dFNC data were computed (first order derivatives of sliding windowed correlations). Lastly, tvFNC data was formed as previously explained.

### Clustering of dFNC and tvFNC Data

The dFNC data was represented by five FNC states using the K-means clustering algorithm along with the correlation distance metric. The optimal number of states/clusters *k* = 5 was identifying using the elbow criterion of the cluster index (Damaraju et al., 2014). To be able to compare our results to the dFNC results, the tvFNC data was clustered with the same cluster algorithm, number of clusters and distance metric. Connectivity dynamism was assessed by two measures computed from the clustering results (1) mean dwell time and (2) fraction time. Mean dwell time provides an average time an individual spend in each state before changing to another state, and fraction time provides a percentage of total time an individual spend in each state.

### Group Differences

Group differences in tvFNC between HC and SZ subjects were tested using two sample *t*-tests and results were corrected for multiple comparisons applying false discovery rate threshold at a significant level of *q* < 0.05. Group differences were tested for connectivity dynamism on the clustering measures, mean dwell time and fraction time; and for FNC states on all FNC pairs. In each state, first we identified subjects with at least one tvFNC element ([wFNC DwFNC]) in that state. Then, the median of all identified tvFNC elements was calculated as the subject state contribution. Therefore, the number of subjects in each state is not fixed. Next, we separate subjects’ states’ median FNC as states FNC and their corresponding derivatives. Lastly, SZ-HC group differences were tested in each state and their corresponding derivatives for each FNC pair.

## Results

### Simulated dFNC Data

Simulations were designed to extend three original FNC states (Figure 2A) into five states (Figure 2D). Dynamically, the first two states show two patterns of positive and negative derivatives, and the last state show small connectivity changes across time (Figure 2E). From the Elbow plot Figure 2C, we can observe that the dFNC method shows a sharp decay in the cluster index from two to three clusters. This result could imply that three could be selected as the optimal number of clusters/states. However, we can notice no changes in the cluster index for the number of clusters bigger than five. In other words, this data can be well represented by five clusters. On the other hand, the tvFNC method shows smooth cluster index decay from two to five clusters and small decline for the higher number of clusters. The tvFNC clustering results with the optimal number of clusters, *k* = 5 are shown in Figure 2D (states) and Figure 2E (states derivatives). These results show that the inclusion of a derivative pattern in the simulation allowed for a clearer identification of similar clusters with different temporal behavior.

The tvFNC method supports the identification of very similar states capturing different temporal behavior not shown in the dFNC method. The absence of derivatives in the clustering estimation resulted in a poor differentiation of similar states even at small noise perturbations. As in the simulation, clustering of real data analyzed in the next subsection can also benefit from the extra information provided by the derivatives.

### Resting State fMRI Data

**FIGURE 3:**
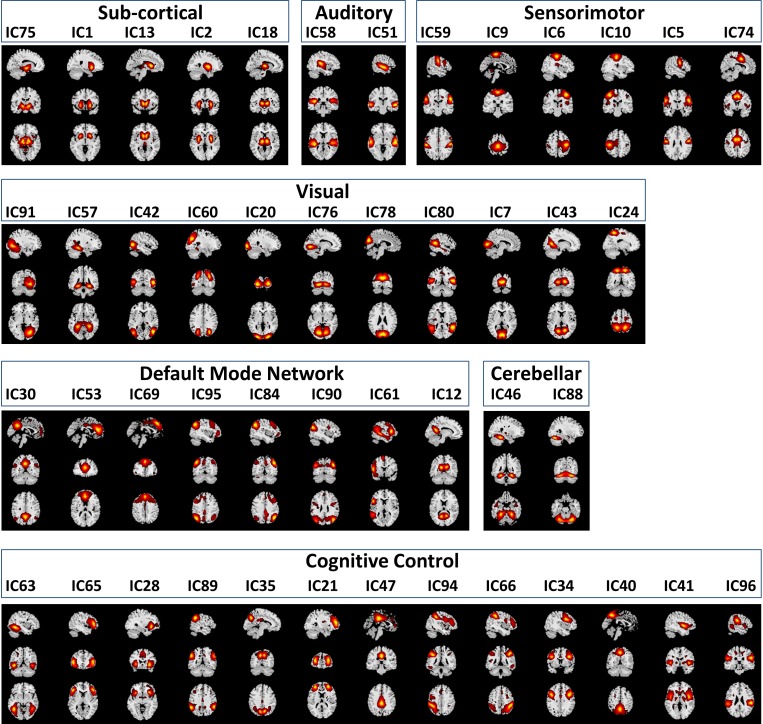
Spatial Maps and their corresponding independent component numbers of the 47 selected resting state networks group into seven domains subcortical (SC), auditory (AUD), sensorimotor (SM), visual (VIS), default mode network (DMN), cerebellar (CB), and cognitive control (CC).

Functional classification of the selected 47 RSNs is based on anatomy and brain functioning. The 47 RSNs were grouped into seven functional domains: sub-cortical [(SC), 5 RSNs]; auditory [(AUD), 2 RSNs]; visual [(VIS), 11 RSNs]; sensorimotor [(SM), 6 RSNs]; attention/cognitive control [(CC), 13 RSNs]; default mode network [(DMN), 8 RSNs]; cerebellar [(CB), 2RSNs]. Table 1 in Damaraju et al. (2014) of the 47 RSNs along with their Brodmann area numbers, number of voxels in each components cluster, component numbers and peak activation coordinates x, y, and z. Figure 3 depicts the spatial maps of the 47 RSNs grouped by seven functional domains.

### Temporal Variation of Functional Network Connectivity Characterization

**FIGURE 4:**
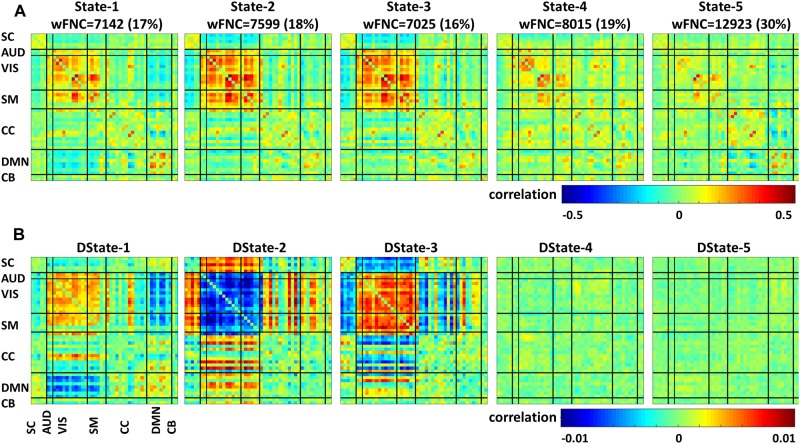
Functional network connectivity states **(A)** and their derivatives **(B)**.

Using sliding-window and k-means clustering whole brain temporal variation of FNC during scan duration were represented by five connectivity states. Figure 4 displays the centroids of the five states broke down as FNC states 1–5 (first row) and their corresponding derivatives (second row); the total number of wFNC in each state along with its equivalent percent frequency in parenthesis is also listed. States numbering was assigned based on the order of clustering formation. States’ FNC pattern description is presented in descend order based on their wFNC frequency distribution.

State 5, the one with the higher frequency distribution (30% wFNC) shows weak connectivity overall. This state displays mostly negative correlations between domains and positive correlations within visual, cognitive control and default mode domains. The derivative of State 5 is represented by an unstructured weak connectivity pattern. Small positive and negative correlation values display a mix of increasing and decreasing connectivity.

State 4, the one with the second higher frequency distribution (19% wFNC) shows slightly stronger connectivity than State 5, with higher within- and between connectivity modularity in the visual and sensorimotor domains. The derivative of State 4 is represented by a similar unstructured weak connectivity as the derivative of State 5. In addition, this state derivative displays more pronounced positive correlation values between cognitive control and visual domain can be observed.

State 2, the one with the third higher frequency distribution (18% wFNC) shows a more structured connectivity pattern compared to States 4 and 5. This state captures stronger positive correlations within the visual and sensorimotor domains, and between most RSNs from the auditory, visual, and sensorimotor domains, and a few components from the cognitive control and default mode domains. We can also observe notable negative correlations between the subcortical domain and the auditory, visual, and sensorimotor domains. The derivative of State 2 is represented by a well-structured connectivity pattern displaying increase and/or decrease in connectivity within and between domains. We can observe that the marked positive and negative FNC captured in State 2 has negative and positive derivatives. These results imply that (1) there is noticeable decrease in connectivity within the visual domain and between the auditory, visual, sensorimotor and a few components from the cognitive control domains. (2) There is noticeable increase in connectivity between the subcortical, auditory, sensorimotor and some components from the cognitive control and default mode domains.

State 1, the one with the second lowest frequency distribution (17% wFNC) shows weaker connectivity patterns than States 2 and 3. This state also shows noticeable positive correlations between RSNs from the default mode domain. In addition, we can observe slightly stronger negative correlations between the auditory, visual, sensorimotor, and default node domains. The derivative of State 1 is represented by weaker connectivity pattern in comparison to the derivative of State 3.

We can observe that most of the pronounced positive and negative FNC captured in State 1 has positive and negative derivatives. These results imply that (1) there is a noticeable increase in connectivity within the visual domain and between the auditory, visual, sensorimotor and a few components from the cognitive control domains. (2) There is a noticeable decrease in connectivity between the auditory, visual, and default mode domains. Also we can observe weaker decrease in connectivity between the subcortical, cognitive control, and cerebellar and the rest of domains.

State 3, the one with the lowest frequency distribution (16% wFNC) shows very similar connectivity patterns as State 2. However, the derivative of State 3 is represented by a well-structured connectivity pattern very different than the derivative of State 2. The derivative of State 3 seems like the complement of the derivative of State 2 displaying increase and/or decrease in connectivity within and between domains. We can observe that the marked positive and negative FNC captured in State 3 has positive and negative derivatives. These results imply that (1) there is a noticeable increase in connectivity within the visual domain and between the auditory, visual, sensorimotor and a few components from the cognitive control and default node domains. (2) There is a noticeable decrease in connectivity between the subcortical, auditory, sensorimotor and some components from the cognitive control and default mode domains.

**FIGURE 5:**
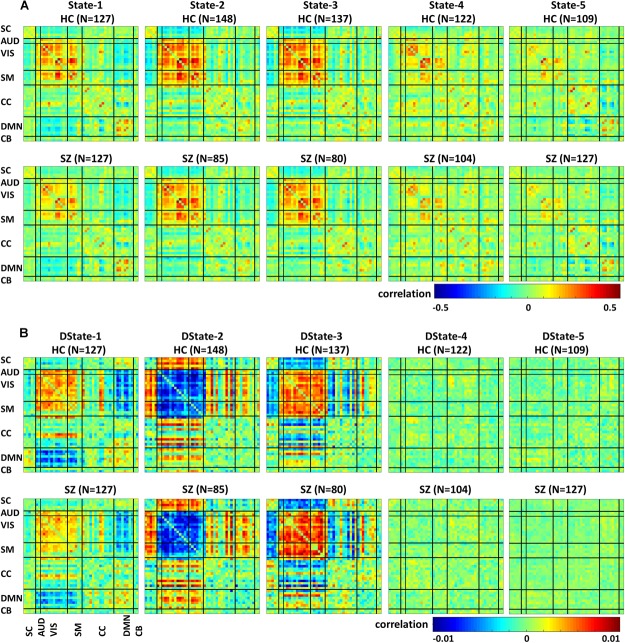
Healthy control (HC) and schizophrenia (SZ) participants’ functional Network connectivity states **(A)** and their derivatives **(B)**.

Figure 5 depicts the FNC states (A) and their derivative (B) centroids separated by groups HC (first row) and SZ (second row). The total number of subjects in each state is listed in parenthesis. The HC and SZ group FNC states 1–5 and their derivatives patterns are very similar to those shown in Figure 4. State 1, the fourth state in the wFNC percent frequency rank contains the highest number of subjects [*N* = 254, HC = 127, and SZ = 127]. It is followed by State 5 [*N* = 236, HC = 109, and SZ = 127], the number one in the wFNC percent frequency rank. The HC FNC states show slightly higher positive and negative connectivity patterns than SZ states.

### Schizophrenia and Healthy Control Group Differences in Temporal Variation of Functional Network Connectivity

**FIGURE 6:**
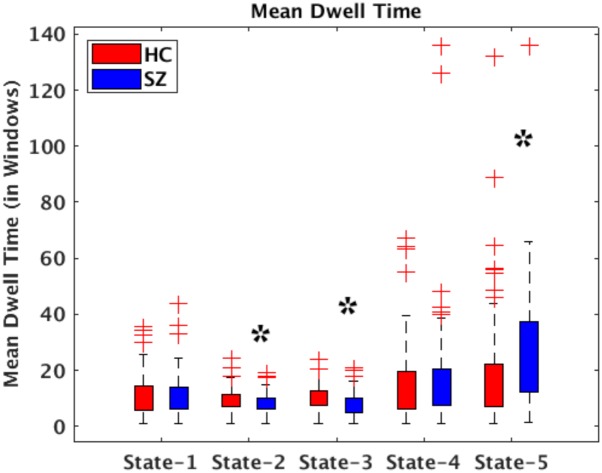
Bar plots displaying mean dwell times in States 1–5 for HC (red) and SZ (blue) participants. The states showing significant differences between the HC and SZ groups are marked with a black star (FDR-corrected results). The two test *t*- and *p*-values are listed in Table 1.

All presented results were corrected for multiple testing. From Figure 6 we can observe that HC individuals spend significantly more time in States 2 and 3. These states show stronger within- and between-connectivity in the auditory, visual, and sensorimotor domains compared to the other states. On the other hand, SZ individuals spend more time in State 5 (a state displaying weakly connectivity between RSNs from all domains). The *t*- and *p*-values are listed in Table 1.

**Table 1:** Two *t*-test mean dwell time and fraction time results showing Healthy control (HC) and Schizophrenia (SZ) differences in each state.

	State-1	State-2	State-3	State-4	State-5
**Mean dwell time**					
*p*-value	0.6984	0.0278^∗^	0.0058^∗^	0.3811	9.88e – 05^∗^
*t*-Value	−0.3880	2.2134	2.7899	−0.8776	−3.9618
**Fraction time**					
*p*-value	0.1089	0.0006^∗^	3.22e – 07^∗^	0.9943	9.98e – 10^∗^
*t*-Value	−1.6088	3.4697	5.2759	−0.0072	−6.3690

*The FDR corrected p-values showing significant differences between HC and SZ are marked with a star.*

**FIGURE 7:**
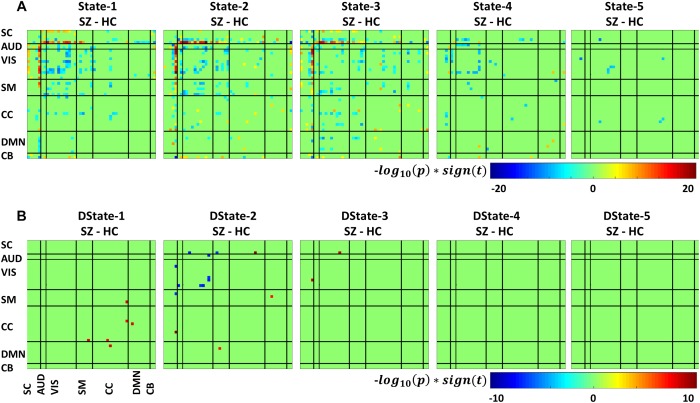
Two *t*-test results showing states **(A)** and derivatives **(B)** connectivity differences between the SZ and HC groups, FDR corrected results threshold at a *q* < 0.05.

Figure 7 depicts the significant connectivity differences between SZ and HC subjects in states 1–5 (Figure 7A, first row) and in the states derivatives (Figure 7B, second row). From Figure 7A, showing FNC group differences in states 1–3 we can observe that compared to HC, SZ patients showed higher connectivity between a RSN from the subcortical domain [thalamus (IC18)] and RSNS from the auditory [superior temporal (IC58) and middle temporal gyrus (IC51)], visual [lingual gyrus (IC91), parahippocampal gyrus IC(57), middle temporal gyrus(IC42), middle frontal gyrus (IC20), cuneus (IC78), middle temporal gyrus (IC80), cuneus IC(7), superior parietal lobule (IC24)], and sensorimotor [medial frontal gyrus (IC9), right post-central gyrus (IC6)] domains. We can also notice less pronounced connectivity between RSNs from the subcortical domain and RSNs from the auditory, visual, cognitive control, and cerebellar domains in State 1; between RSNs from the subcortical and cerebellar domains and RSNs from the other domains in States 2 and 3; between subcortical, auditory, visual, cognitive control, default mode, and cerebellar domains in State 4. On the other hand, compared to SZ, HC showed higher within connectivity in the visual, sensorimotor, cognitive control domains; and among the subcortical, auditory, sensorimotor and the rest of domains. From Figure 7B, we can observe FNC group differences between SZ and HC in the derivatives of states 1–3. No significant differences in the derivatives of states 4 and 5 were found. Compared to HC, SZ subjects showed higher increase in derivatives in State 1 between inferior parietal lobe (IC96, CC) and precentral gyrus (IC5, SM), middle frontal gyrus (IC21, CC); and between cingulate gyrus (IC47, CC) and anterior cingulate gyrus (IC53, DMN); in State 2 between inferior frontal gyrus (IC34, CC) and thalamus (IC18, SC), and middle frontal gyrus (IC69, DMN) and post-central gyrus (IC6, SM); and in State 3 between middle temporal gyrus (IC80, VIS), and thalamus (IC18, SC).

## Discussion

In this work we have presented the tvFNC method which is an extension of the current dFNC approach to include the first derivative of the time dependent FNC patterns in the overall analysis. We found that time derivatives exhibits their own clustering patterns. The inclusion of the derivative information was useful for the clustering procedure to find an accurate clustering partition.

### Clustering and tvFNC

Simulated data showed that the identification of occurring connectivity patterns performed by clustering analysis can benefit from using the first derivative information to support the existence of similar patterns with different temporal behavior. We confirm the premise that time point information can be better described, and subsequently clustered, by using its derivative. It is not the first time that derivatives are used to improve the characterization of a time varying signal. This assumption is rooted in Taylor’s theorem. Notice that due to fMRI protocols we don’t really have a continuous description of the signal. In fact, the fMRI data consists of snapshots spaced in time by a predefined TR. In the current context, we are improving the discrete time estimation of the dFNC at a given time point t = n^∗^TR by adding information of the estimated derivative DdFNC at that time point. The simulation showed that including time specific estimations of derivatives helped in recognizing the different dFNC patterns imposed in the simulation. The importance of the derivative extends to the real data where an extra set of observations can be accounted for.

Dynamic FNC was captured by five connectivity states that reoccurred over time supporting previous finding that whole-brain functional connectivity is not stationary (Allen et al., 2014; Damaraju et al., 2014; Rashid et al., 2014; Faghiri et al., 2018; Espinoza et al., 2019). In addition, the dFNC states’ time derivatives provide a measure that is sensitive to dFNC changes over a period of time. These tvFNC results are also in line with previous resting state studies results examining functional disruptions in SZ. For instance, the five dFNC states identified in this study are very similar to those identified by Damaraju and collaborators (Damaraju et al., 2014). In that study, the optimum number of clusters representing connectivity states was selected using the Elbow criterion. Based on this approach, five states were obtained to describe FNC over time. It can be observed in both, Figure 4 here and in Damaraju et al., that States 2 and 3 are very similar. From just looking at the dFNC states results, it can be inferred that the number of FNC states can be reduced from five to four. However, the derivative patterns observed using the tvFNC method complements the results from the dFNC approach, validating the previously obtained FNC states and providing additional support for states separation. A clear state differentiation is observed from the FNC derivatives of States 2 and 3 which display different connectivity patterns, Figure 4B.

Another important observation to make is that the connectivity patterns of States 2 and 3 derivatives seem to complement each other. For example, from Figure 4 we observe that the derivative of State 2 shows decreasing connectivity among auditory, visual and sensorimotor domains while the derivative of State 3, shows increasing connectivity among these domains. On the other hand, the derivative of State 2 shows increasing connectivity among subcortical, auditory, visual, and sensorimotor domains while the derivative of State 3 shows decreasing connectivity among these domains. Overall, both states derivatives values are very close to zero showing almost constant (very small temporal variations) connectivity over time.

### HC Versus SZ

In terms of dynamism, HC changed states more than SZ subjects did. These changes were measured by computing the fraction time (FT) spend in states for the two groups. Compared to HC, SZ individuals spend significant more time in State 5, a state showing weakly dFNC and almost constant behavior over time. Lower degree of functional connectivity and reduced in modularity in SZ was also reported by Lynall et al. (2010), Yu et al. (2011), and Damaraju et al. (2014). The tvFNC analysis captured group differences in all five states. It also uncovered significant group differences in States 4 and 5 not previously captured (Damaraju et al., 2014). Figure 7 shows SZ individuals with lower connectivity than HC in states where the connectivity is already weak (States 4 and 5). These two states might be visualized as a valley or a point in time where the general connectivity lowers then rises. Since there is no significant difference in the derivatives of States 4 and 5, we can argue that spending more time in the weak states (just as SZ subjects do) allows reaching lower connectivity. On the contrary, HC dwelling is shorter and the connectivity does not reach the same minimum value. This new observation shows extra evidence that derivatives gives new refinements in the analysis. With respect to States 1, 2, and 3, Figure 7A shows hyperconnectivity in SZ between the subcortical (thalamus) and RSNs from the auditory, visual and sensorimotor domains. Hypoconnectivity between (1) subcortical and cognitive control and default mode domains; (2) default mode and cognitive control domains; and (3) cerebellar and auditory, visual and sensorimotor, cognitive control domains is also observed. These states (1, 2, and 3) suffer more sudden and faster changes, thus the dynamics are different than States 4 and 5. Note that, a similar pattern of hypoconnectivity within auditory and visual domains can be seen in all five states which is consistent with the disconnection hypothesis (Friston et al., 2016). Results seem in higher agreement with the disconnection hypothesis since some dFNC has slower dynamicity as seen in Figure 5B, 6 where some derivatives exhibit lower magnitudes in SZ.

In a compensatory manner, thalamic connectivity is stronger in SZ as it is the main characteristic shared by states 1, 2, and 3. Although this observation seems contrary to Friston’s disconnection hypothesis, it is not a rare observation. Resting-state fMRI studies have reported SZ thalamic hyperconnectivity with sensorimotor cortices, whole brain (Woodward et al., 2012; Damaraju et al., 2014; Rashid et al., 2014) and seed-based (Woodward et al., 2012; Anticevic et al., 2014). To counterbalance the previous statement, we must point out that thalamic hyperconnectivity pertain to states with short dwelling while larger dwelling states characterizes the absence of this hyperconnectivity (see Figure 5, 6). However, sensorial hypoconnectivity (auditory, visual and sensorimotor) can be found in all states, thus present 100 % of the time.

The novel contributions of this work are the differences in dynamicity, as measured using the derivatives of dFNC. Figure 7B displays states derivative differences’ between SZ and HC. Group differences were captured in three out of the five states among six domains. Compared to HC, SZ subjects showed higher increase in derivatives in State 1 between inferior parietal lobe and, precentral gyrus and middle frontal gyrus; and cingulate gyrus and anterior cingulate gyrus. In State 2 between inferior frontal gyrus and thalamus; and middle frontal gyrus and post-central gyrus. In State 3 between middle temporal gyrus and thalamus. Despite these increments in variation, the connectivity strength was not different for the mentioned IC pairs in States 2 and 3. This can be observed by comparing the mentioned derivatives in Figure 5B with the connectivity in Figure 5A. This observation is not consistent since we could expect that higher derivatives would help increasing the magnitude of connectivity. Since this was not the case, we can conclude that the aggregated effect of the increased derivatives was not coherent or not strong enough to produce a consistent difference in connectivity. However, the observation points to a more rapid connectivity fluctuation in CC and DMN brain areas pointing to possible causes of cognitive deficiencies known to occur in schizophrenia (Schaefer et al., 2013).

### Limitations and Future Directions

Among the limitations to be consider in this work: Functional connectivity is measured as the Pearson correlation between fMRI time-courses, and this lower order statistic provided significant results. Higher order statistics, such as mutual information, could be considered in future work to extend this analysis. The calculation of dFNC data requires a window length selection. The selected windowed size should be able to capture functional connectivity variability in small time domains (Sakoğlu et al., 2010). Following this recommendation, a fixed window size of 22 TR (=44 s) similar to the one used in Damaraju et al. (2014) was selected. Future work should evaluate state derivative changes over range of window sizes. Another limitation lies on the scan duration. This resting state fMRI data was collected for 5.4 min, a longer scanning time may uncover the identification of critical time points where FNC states reaches stationary behavior.

## Ethics Statement

Participants’ scan and information collection was approved by all seven sites’ institutional review boards (IRB). Each participant provided written informed consent before scanning.

## Author Contributions

FE, VV, and VC designed the methods and carried out the study. VV and AF helped to refine the data simulations. FE, ED, and KH carried out the data analysis. JT, AB, JF, SM, DM, BM, SP, AP, JV, and TvE collected the data. FE wrote the first draft of the manuscript. All authors contributed to the manuscript revision, read, and approved its final version.

## Conflict of Interest Statement

The authors declare that the research was conducted in the absence of any commercial or financial relationships that could be construed as a potential conflict of interest.

## Footnotes

1 http://mialab.mrn.org/software/gift/

2 https://afni.nimh.nih.gov/

3 http://www.fil.ion.ucl.ac.uk/spm/
